# Does washing insecticide-treated nets 20 times for experimental hut evaluations provide a suitable proxy for their end-of-life performance under household conditions?

**DOI:** 10.1186/s13071-025-06743-w

**Published:** 2025-04-21

**Authors:** Thomas Syme, Abel Agbevo, Josias Fagbohoun, Boris N’dombidjé, Judicael Nounagnon, Juniace Ahoga, Joël Akpi, Corine Ngufor

**Affiliations:** 1 London School of Hygiene and Tropical Medicine (LSHTM), London, WC1E 7HT UK; 2 Centre de Recherches Entomologiques de Cotonou (CREC), Cotonou, Benin; 3 Pan-African Malaria Vector Research Consortium (PAMVERC), Cotonou, Benin

**Keywords:** Insecticide-treated nets, Long-lasting insecticidal nets, Malaria, Vector control, Mosquitoes

## Abstract

**Background:**

Insecticide-treated nets (ITNs) are washed 20 times as part of experimental hut trials to simulate the loss of active ingredient (AI) occurring over their intended 3-year lifespan and estimate insecticidal durability. The ability of the 20-wash method to predict the end-of-life performance of ITNs has not been empirically validated.

**Methods:**

We performed an experimental hut trial to compare the efficacy of new ITNs unwashed and washed 20 times to field-aged ITNs withdrawn from households 3 years post-distribution against a pyrethroid-resistant vector population in Covè, Benin. Four products from pyrethroid-only (Interceptor^®^), pyrethroid-piperonyl butoxide (PermaNet^®^ 3.0), pyrethroid-pyriproxyfen (Royal Guard^®^) and pyrethroid-chlorfenapyr (Interceptor^®^ G2) ITN types were tested. Net pieces were tested in bioassays and sent for chemical analysis to assess differences in surface AI bioavailability and total chemical content between washed and field-aged nets. Susceptibility bioassays were also performed to assess insecticide resistance in the Covè vector population.

**Results:**

Mosquito mortality in experimental huts was similar or slightly higher with field-aged nets than washed nets with Interceptor^®^ (11% vs. 10%, *p* = 0.339, OR = 1.19, 95% CIs [0.84, 1.69]), PermaNet^®^ 3.0 (12% vs. 18%, *p* < 0.001, OR = 1.78, 95% CIs [1.34, 2.38]) and Royal Guard^®^ (9% vs. 14%, *p* = 0.076, OR = 1.33, 95% CIs: [0.97, 1.83]). Likewise, field-aged Royal Guard^®^ induced a similar reduction in fertility to washed Royal Guard^®^ (22% vs. 29%, *p* = 0.066). In contrast, mortality was significantly lower with field-aged nets Interceptor^®^ G2 compared to washed nets (54% vs. 19%, *p* < 0.001, OR = 0.18, 95% CIs [0.14, 0.24]). Blood-feeding inhibition was higher with field-aged nets than washed nets across all ITN types. Retention of non-pyrethroid AIs was lower than for the pyrethroid, particularly with field-aged nets (PermaNet^®^ 3.0 (roof): 25% vs. 68%, *p* < 0.001, Royal Guard^®^: 27% vs. 53%, *p* < 0.001, Interceptor^®^ G2: 14% vs. 39%, *p* < 0.001).

**Conclusions:**

In this setting, the 20-wash method provided a suitable proxy for the end-of-life killing and sterilising performance of Interceptor^®^, PermaNet^®^ 3.0 and Royal Guard® in experimental huts. In contrast, washing overestimated the end-of-life performance of Interceptor^®^ G2 for mortality and underestimated the personal protection of all field-aged ITNs.

**Graphical Abstract:**

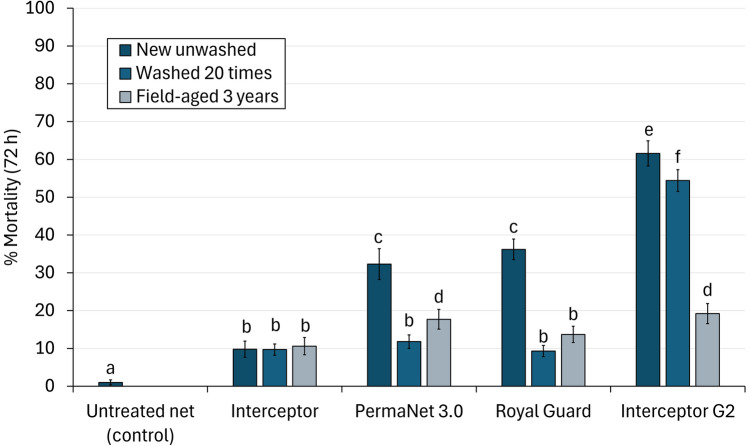

**Supplementary Information:**

The online version contains supplementary material available at 10.1186/s13071-025-06743-w.

## Background

The scaling up of insecticide-treated net (ITN) coverage has been instrumental in reducing malaria transmission and burden globally over the past 2 decades. The proportion of the population in sub-Saharan Africa protected by an ITN rose from < 2% in 2000 to 55% in 2015 [1] and modelling studies estimate that this expansion prevented 451 million malaria cases [2]. Recently, however, declines in malaria cases and deaths have stalled, and the effectiveness of ITNs is threatened on several fronts [3]. Levels of ITN access and coverage, for example, remain inadequate and increasing evidence documents the suboptimal durability of ITNs in many settings [4–6]. Vector resistance to the insecticides used to treat nets, notably pyrethroids, is also increasing [7] and may already be compromising control. To respond to these threats, vector control product manufacturers are developing new ITN products with claims of improved efficacy against insecticide-resistant mosquito vectors.

To be added to the WHO list of prequalified vector control products [8]—and thereby eligible for procurement by major malaria control agencies—new ITN brands must be assessed by the Prequalification Unit Vector Control Product Assessment Team (PQT/VCP) for their safety, quality and efficacy according to established guidelines [9]. To demonstrate entomological efficacy against free-flying mosquitoes, ITNs are subjected to semi-field trials, which assess their biological activity under simulated user conditions [10]. Experimental hut trials are considered the gold standard for assessing the entomological efficacy of ITNs, as they provide data on wild malaria vector populations interacting with a human host under controlled field conditions that recreate the natural interactions between mosquitoes and ITNs inside households [11, 12]. Indeed, recent modelling studies have demonstrated the capacity of these assays to generate entomological endpoints that serve as suitable surrogates for the epidemiological impact of ITNs, as measured in cluster RCTs [13, 14]. The WHO has also recommended experimental hut studies to generate comparative efficacy data of second-in-class ITN brands to first-in-class ITN products for established ITN intervention classes with empirical evidence of public health value [15]. This approach provides a relatively easy and cost-effective means of determining whether an existing WHO recommendation for a given ITN intervention class can be applied to a new ITN product, helping inform procurement decisions by WHO member states.

To be considered long lasting, ITNs are expected to remain effective for at least 3 years under user conditions [16]. During operational use, active ingredient (AI) is removed from the surface of ITNs because of washing and other factors, causing a reduction in the quantity of AI bound in the reservoir over time [17]. The insecticidal durability of ITNs—their ability to retain an adequate level of surface AI and induce intended entomological effects over their lifespan—is thus a critical factor in determining the effectiveness and cost-effectiveness of ITNs and helps guide procurement decisions by control programmes. To evaluate ITN wash resistance in experimental hut trials, the WHO recommends a standardised procedure whereby nets are artificially aged by washing them in soap solution 20 times to simulate the loss of AI occurring over 3 years of operational use [18]. This method was developed based on two main assumptions: (ii) washing is the primary driver of AI loss under operational conditions and (ii) users wash nets approximately once every 2 months.

Previous studies showed that washing reduces both the chemical content and entomological efficacy of ITNs [19, 20]. Washing removes the bioavailable fraction of AI on the net surface, and over repeated washes, this depletes the total chemical content of ITNs, causing reductions in their potency against mosquitoes. While washing nets for hut trials provides a simple, standardised procedure, which ensures consistency and comparability across different studies and products, it remains unclear whether it provides a suitable proxy for the end-of-life performance of ITNs under real-world conditions. Studies are thus needed to empirically validate the use of the 20-wash protocol as a proxy for the end-of-life performance of ITNs and improve estimates of insecticidal wash resistance generated in experimental hut trials.

To assess whether washing ITNs 20 times for hut trials provides a suitable proxy for their end-of-life performance, we performed an experimental hut trial comparing the entomological efficacy and chemical content of new ITNs unwashed and after 20 standardised washes to field-aged ITNs withdrawn from households in Benin 3 years post-distribution against a pyrethroid-resistant vector population in southern Benin. Four WHO-prequalified ITN products from all intervention classes included under current WHO policy recommendations were tested, including pyrethroid-only (Interceptor^®^), pyrethroid-PBO (PermaNet^®^ 3.0), pyrethroid-PPF (Royal Guard^®^) and pyrethroid-CFP (Interceptor^®^ G2) nets. Net pieces cut from ITNs used in the hut trial were tested in supplementary laboratory bioassays and sent for chemical analysis to characterise the bioavailability of AIs on the net surface and measure changes in total AI content after artificial and operational ageing. Susceptibility bioassays were also performed during each trial to assess phenotypic resistance of the local vector population to the AIs in the study ITNs and support interpretation of the experimental hut results.

## Methods

### Experimental hut trials

To assess the ability of the 20-wash method to simulate the end-of-life performance of ITNs, we performed an experimental hut trial comparing the entomological efficacy of ITNs washed 20 times to field-aged ITNs withdrawn after 3 years of operational use against wild, free-flying malaria vectors.

#### Study site and vector population

The experimental hut trials were performed at Centre de Recherche Entomologique de Cotonou and London School of Hygiene & Tropical Medicine (CREC/LSHTM) field site in the commune of Covè, Zou Department, southern Benin (7°14′N2°18′E). The huts are surrounded by rice paddies, which provide permanent and extensive breeding sites supporting a high year-round density of malaria vectors. *Anopheles coluzzii* and *An. gambiae* s.s. occur sympatrically with the former predominating [21]. Recent studies characterising the insecticide resistance profile of the Covè vector population using susceptibility bioassays have demonstrated a high frequency and intensity of pyrethroid resistance but continued susceptibility to other insecticides, including CFP and PPF [22, 23]. Pre-exposure to PBO improves the mortality response to pyrethroids without restoring full susceptibility, demonstrating the partial contribution of cytochrome P450 monooxygenases (P450s) to pyrethroid resistance [23]. This is corroborated by genotyping and gene expression studies, which reveal a high frequency of the knockdown resistance (*kdr*) mutation (89%) and four-fold overexpression of P450s, including CYP6P3 [21]—a validated marker for metabolic pyrethroid resistance [24]. The experimental huts used were of standard West African design made of concrete bricks with cement-plastered walls enclosed with a corrugated iron roof and polyethene ceiling. Mosquitoes entered via four window slits on the front and side walls of the hut. Each hut had a wooden-framed veranda projecting from the rear wall to capture exiting mosquitoes and was surrounded by a water-filled moat to prevent entry of scavenging ants.

#### Experimental hut treatments

An untreated control net and four WHO-prequalified ITN products included under current WHO policy recommendations were used for the study. The specifications of the study ITNs are described below:

i. Interceptor^®^ (BASF) is a standard pyrethroid-only ITN made of 100-denier polyester filaments coated with 5 g/kg (200 mg/m^2^) of alpha-cypermethrin. Its dimensions are 1.8 (L) × 1.8 (W) × 1.8 (H) m. The fabric weighs 40 g/m^2^ and has a minimum bursting strength of 405 kPa.
ii. PermaNet^®^ 3.0 (Vestergaard Sàrl) is a pyrethroid-PBO ITN. It features a mosaic design consisting of 100-denier polyester side panels coated with 2.1 g/kg (84 mg/m^2^) of deltamethrin and a 100-denier polyethylene roof panel incorporated with a mixture of deltamethrin and PBO at 4 g/kg (120 mg/m^2^) and 25 g/kg (800 mg/m^2^) respectively. Its dimensions are 1.6 (L) × 1.8 (W) × 1.5 (H) m. The fabric weight and minimum bursting strength are 40 g/m^2^ and 350 kPa on the sides compared to 30 g/m^2^ and 400 kPa on the roof.
iii. Royal Guard^®^ (Disease Control Technologies) is a pyrethroid-PPF ITN made of 120-denier high-density polyethylene filaments incorporated with a mixture of alpha-cypermethrin and PPF at target doses of 5.5 g/kg (208 mg/m^2^) each. Its dimensions are 1.8 (L) × 1.8 (W) × 1.6 (H) m, while its fabric weighs 38 g/m^2^ and has a minimum bursting strength of 350 kPa.
iv. Interceptor^®^ G2 (BASF) is a pyrethroid-CFP ITN made of 100-denier polyester filaments coated with a mixture of alpha-cypermethrin and CFP at target doses of 2.4 g/kg (100 mg/m^2^) and 4.8 g/kg (200 mg/m^2^) respectively. Its dimensions are 1.8 (L) × 1.8 (W) × 1.8 (H) m, while its fabric weighs 40 g/m^2^ and has a minimum bursting strength of 405 kPa.

The experimental hut trial compared the entomological efficacy of new ITNs unwashed and after 20 standardised washes to field-aged ITNs withdrawn from households after 3 years of operational use. A total of 6 new unwashed and washed replicate nets and 78 field-aged replicate nets were tested and rotated within the treatments daily. A larger sample of field-aged nets was used to help control for variation in net care and use practices between users, which may affect their entomological efficacy and bias experimental hut results. The following 13 treatment arms were thus evaluated in experimental huts:

1. Untreated net (control): 6 replicate nets
2. Interceptor^®^ (new unwashed): 6 replicate nets
3. Interceptor^®^ (washed 20 times): 6 replicate nets
4. Interceptor^®^ (field-aged 3 years): 78 replicate nets
5. PermaNet^®^ 3.0 (new unwashed): 6 replicates
6. PermaNet^®^ 3.0 (washed 20 times): 6 replicates
7. PermaNet^®^ 3.0 (field-aged 3 years): 78 replicates
8. Royal Guard^®^ (new unwashed): 6 replicates
9. Royal Guard^®^ (washed 20 times): 6 replicates
10. Royal Guard^®^ (field-aged 3 years): 78 replicates
11. Interceptor^®^ G2 (new unwashed): 6 replicates
12. Interceptor^®^ G2 (washed 20 times): 6 replicates
13. Interceptor^®^ G2 (field-aged 3 years): 78 replicates

#### Preparation of study nets

New nets were tested unwashed and washed 20 times to simulate end-of-life performance. Nets were washed according to WHO guidelines [18]. The nets were submerged in an aluminium bowl containing 2 g of Savon de Marseille dissolved in 10 l water and washed for 10 min. After washing, nets were rinsed twice in 10 l clean water following the same procedure and dried horizontally in the shade. The time interval applied between washes for each ITN product was selected based on the regeneration times outlined in WHO assessment reports as follows: 1 day for Interceptor^®^, PermaNet^®^ 3.0 and Interceptor^®^ G2 and 3 days for Royal Guard^®^ [25–28]. To simulate wear and tear from regular use, the untreated control nets and new unwashed and washed ITNs were given six holes measuring 4 × 4 cm, one on each short side panel and two on each long side panel. Field-aged nets withdrawn after 3 years were obtained from ongoing durability monitoring studies nested within a cluster RCT designed to evaluate the epidemiological efficacy of next-generation ITNs compared to standard pyrethroid-only nets in Benin. The design of the durability monitoring study has been described previously [29]. To support the interpretation of the experimental hut results, the hole index of field-aged nets was also calculated by counting and classifying the number of holes according to their location and size as per WHO guidelines [18]. Nets were erected inside huts by tying the four corners of the roof panel to nails positioned in the top corners of the hut.

#### Experimental hut trial procedure

Consenting human volunteers slept in experimental huts from 21:00 to 06:00 to attract wild, free-flying mosquitoes. Treatments were rotated between huts weekly, while volunteers were rotated daily according to Latin square designs to mitigate variation due to differences in positional and host attractiveness to mosquitoes. Each morning, volunteers collected all mosquitoes from the different compartments of the hut (under the net, room and veranda) and deposited them in labelled holding cups using a torch and aspirator. Mosquito collections were transferred to the field laboratory for morphological identification and scoring of immediate mortality (live/dead) and blood-feeding (unfed/blood-fed). Live female *An. gambiae* s.l. were retained and provided access to cotton wool soaked in 10% (w/v) glucose solution. Delayed mortality was recorded every 24 h up to 72 h after collection, and % 72 h mortality was used as the primary mortality endpoint for all treatments to account for the delayed action of CFP [30]. To evaluate the impact of Royal Guard® on fecundity, subsamples of surviving blood-fed *An. gambiae* s.l. were dissected to observe ovary development and score fertility as previously described. Mosquito collections were performed 6 days per week for one full treatment rotation (13 weeks, 78 nights) during the long rainy season between May and July 2023. Huts were cleaned and aired on the 7th day to prevent contamination before the next rotation cycle.

#### Experimental hut trial outcome measures

For each treatment, the total number of alive/dead, unfed/blood-fed and fertile/infertile mosquitoes collected in the different hut compartments were pooled to generate a variety of outcome measures (listed below) of entomological efficacy. The primary outcome measures used to compare the performance of ITNs against pyrethroid-resistant *An. gambiae* s.l. after artificial and operational ageing were:

i. Mortality (%): the proportion of dead mosquitoes immediately after collection and every 24 h up to 72 h thereafter.

ii. Blood-feeding inhibition (%): the reduction in the proportion of blood-fed mosquitoes in the treated hut relative to the untreated control hut, calculated as follows:

$$Blood feeding inhibition \left( \% \right) = \frac{{100 \left( {Bfu - Bft} \right)}}{Bfu}$$

iii. Reduction in fertility (%): the reduction in the proportion of dissected mosquitoes scored as fertile for a given treatment compared to the control, calculated as follows:

$$\text{Reduction in fertility (\%)} = \frac{100 \left( Fu - Ft \right)}{Fu}$$

where *Fu* is the proportion of fertile mosquitoes in the untreated control hut, and *Ft* is the proportion of fertile mosquitoes in the treated hut.

The secondary outcome measures used to express the efficacy of the experimental hut treatments against pyrethroid-resistant *An. gambiae* s.l. were:

i. Entry (*n*): the number of mosquitoes collected inside the hut.
ii. Deterrence (%): the reduction in entry in the treated hut relative to the untreated control hut, calculated as follows:
  $$Deterrence \left( \% \right) = \frac{{100 \left( {Tu - Tt} \right)}}{Tu}$$

Here, *Tu* is the number of mosquitoes entering the untreated control hut, and *Tt* is the number of mosquitoes entering the treated hut.

iii. Exophily (%): exiting rates due to the potential irritant effect of treatments expressed as the proportion of mosquitoes collected in the veranda.
iv. Blood-feeding (%): the proportion of blood-fed mosquitoes.
v. Fertility (%): the proportion of dissected mosquitoes scored fertile.

### Preparation of net pieces for supplementary bioassays and chemical analysis

Five net pieces (one from each panel) measuring 30 × 30 cm were cut from randomly selected nets per treatment arm at positions outlined in WHO guidelines [18]. Given its mosaic design, an additional two net pieces were cut from the roof of PermaNet^®^ 3.0 as per WHO specifications [31] to provide a total of seven pieces and a representative sample of pieces incorporated with deltamethrin and PBO. After cutting, net pieces were labelled, wrapped in aluminium foil and stored in an incubator at 30 ± 2 °C before and between testing in laboratory bioassays.

### Supplementary laboratory bioassays

To support the interpretation of the results, net pieces cut from whole unwashed, washed and field-aged nets were tested in supplementary laboratory bioassays to characterise the bioavailability of the key AIs on the surface of the study ITNs. Test methods and mosquito strains were selected to capture the intended biological effect of each AI. To characterise the rapid toxicity of alpha-cypermethrin on Interceptor^®^ and the sterilising effects of PPF on Royal Guard®, cone bioassays were performed respectively with unfed mosquitoes of the susceptible Kisumu strain and blood-fed mosquitoes of the insecticide-resistant Akron strain. Meanwhile, given the well-documented unsuitability of cone bioassays for evaluating ITNs treated with certain insecticides [32], tunnel tests were performed with unfed mosquitoes of the insecticide-resistant Covè and Akron strains to characterise the killing effects of PBO on the roof of PermaNet^®^ 3.0 and CFP on Interceptor^®^ G2. The characteristics of these mosquito strains are described below:

- The *An. gambiae* s.s. Kisumu strain is a susceptible reference strain originating from Kisumu, western Kenya.
- The *An. gambiae* s.l. Covè strain is a pyrethroid-resistant strain, which is the first filial (F1) progeny of mosquitoes collected as larvae near the experimental hut site in Covè, Zou Department, southern Benin. The species composition and resistance profile have been described previously [21].
- The *An. coluzzii* Akron strain is an insecticide-resistant strain from Akron near Porto-Novo in southern Benin. It exhibits resistance to pyrethroids, organochlorines and carbamates mediated by the *kdr* L995F and insensitive acetylcholinesterase (AChE (*ace-1*^*R*^) G119S mutations and elevated activity of P450s and esterases [33, 34].

During all tests, parallel exposures were performed with untreated net pieces as a negative control, and laboratory conditions were maintained at 27 ± 2 °C and 75% ± 10% humidity.

#### Cone bioassays

At least 20 mosquitoes aged 3 to 5 days were exposed in cone bioassays to each net piece for 3 min in four replicates of approximately five mosquitoes. At the end of exposure, mosquitoes were transferred to holding cups, provided access to cotton wool soaked in 10% (w/v) glucose solution and scored for delayed mortality. Mortality after 24 h of the unfed Kisumu strain was selected as the primary endpoint to characterise the rapid action and bioavailability of the pyrethroid on Interceptor^®^. To assess the sterilising effects and bioavailability of PPF in Royal Guard^®^, blood-fed Akron mosquitoes surviving after 72 h were dissected to observe ovary development and score fertility as previously described. Reduction in fertility relative to control was used as the primary endpoint to characterise the sterilising effects of PPF on Royal Guard^®^.

#### Tunnel tests

Tunnel tests are an experimental chamber that simulates the natural behavioural interactions between free-flying mosquitoes and nets during host-seeking. Approximately 100 mosquitoes aged 5 to 8 days were exposed to each net piece overnight in the presence of a guinea pig bait in one replicate test. Nets pieces were given 9 × 1-cm holes to facilitate entry into the baited chamber. In the morning, all mosquitoes were collected from the tunnel and scored for immediate mortality and blood-feeding. Surviving mosquitoes were provided access to 10% (w/v) glucose solution, and delayed mortality was recorded. Mortality after 24 h was used as the primary endpoint to assess the rapid toxicity of synergised pyrethroid on PermaNet® 3.0 (roof), while mortality after 72 h was selected to assess the delayed action of CFP on Interceptor® G2. After the bioassays, net pieces were transferred to a refrigerator and stored at 4 ± 2 °C before being sent for chemical analysis.

### Chemical analysis of net pieces

Net pieces cut from ITNs tested in experimental huts and laboratory bioassays were sent to Centre Walloon de Recherches Agronomiques, Belgium, for chemical analysis to determine within- and between-net variation of AI and retention of AI before and after artificial and operational ageing. The analytical methods used were based on those published by the Collaborative International Pesticides Analytical Council and have been described previously [19, 35].

### Susceptibility bioassays

WHO tube tests and bottle bioassays were performed during the trial to assess the susceptibility of adult *An. gambiae* s.l. collected as larvae from breeding sites near the experimental huts to the AIs in the study ITNs and support interpretation of the results. Tube tests were conducted to assess the frequency and intensity of pyrethroid resistance by exposing mosquitoes to 1 ×, 5 × and 10 × discriminating concentrations of alpha-cypermethrin and deltamethrin (0.05%). Mosquitoes were exposed to 1 ×, 5 × and 10 × the discriminating concentrations of alpha-cypermethrin and deltamethrin (0.05%) in tube tests to assess the frequency and intensity of pyrethroid resistance. PBO synergism and the contribution of P450s to pyrethroid resistance were also investigated by testing the discriminating concentrations of both pyrethroids with pre-exposure to PBO (4%). Susceptibility to CFP and PPF was assessed in bottle assays (100 µg/bottle for each AI). All tests followed WHO guidelines [36]. Proportional mortality after 24 h and 72 h was used to assess susceptibility to the pyrethroids and CFP, respectively. PPF susceptibility, meanwhile, was assessed based on the proportional reduction in fertility among surviving blood-fed mosquitoes, determined using ovary dissection [37], compared to the untreated control.

### Data analysis

For experimental hut trial data, the total number of alive/dead, blood-fed/unfed and fertile/infertile mosquitoes in the different hut compartments was summed for each treatment to calculate proportional means for each outcome with corresponding 95% confidence intervals (CIs). Differences between experimental hut treatments for these proportional outcomes were analysed using logistic regression, while differences in count outcomes (entry) were analysed using negative binomial regression. In addition to the primary explanatory variable of treatment, each model included hut, sleeper, hole index and day as fixed effects to control for variation associated with these factors. Model-derived *p*-values were used to assign compact letter displays denoting the statistical significance of all pairwise comparisons at the 5% level for all outcomes. Adjusted odds ratios (ORs) were also used to compare the impact of washed nets on mosquito mortality after 72 h to field-aged nets for each net type. The regression analyses were performed in Stata 18. Post hoc simulation-based power analyses were performed using the ‘power_calculator_ITN’ function in R version 4.3.2. The estimated statistical power to detect a significant difference (i.e. *p* < 0.05) in mosquito mortality after 72 h between PermaNet^®^ 3.0 washed 20 times and field-aged PermaNet^®^ 3.0 withdrawn from households 3 years post-distribution was 74.2%, 95% CIs [71.4, 76.9].

Primary outcomes from supplementary laboratory bioassays (% mortality, % reduction in fertility) were plotted on graphs to visualise changes in AI surface bioavailability after washing and field-aging. For the chemical analysis data, we performed a one-way analysis of variance followed by a series of post hoc Tukey’s honest significant difference tests to compare total AI content between condition categories (new unwashed, washed 20 times, field-aged 3 years) for each net type. Proportional AI retention in washed and field-aged nets was also calculated relative to new unwashed nets. For next-generation nets containing two AIs, t-tests were conducted to assess whether there was a significant difference in the retention of the pyrethroid compared to the non-pyrethroid. This analysis was performed separately for washed nets and field-aged nets. The susceptibility of the Covè vector population was interpreted based on observed mortality and fertility rates in tube and bottle bioassays according to WHO guidelines [27]. Control mortality was consistently low (< 5%) in experimental hut trials, susceptibility bioassays and supplementary laboratory bioassays; thus, adjustment of test mortality using Abbott’s formula was unnecessary.

## Results

### Experimental hut results

#### Entry, exiting and inside net results of wild pyrethroid-resistant malaria vectors

A total of 12,968 *An. gambiae* s.l. were collected during the trial, corresponding to an average of approximately 13 mosquitoes per treatment per night (Table 1). While some treatments significantly reduced entry compared to the control, the Royal Guard^®^ and washed ITN arms induced negative deterrence. Proportional exiting was 41% with the untreated control net, and all ITN treatments induced significantly higher exiting than the control (*p* < 0.001). The highest exiting rates were generally observed with PermaNet^®^ 3.0 and Royal Guard^®^ regardless of net condition. For all ITN types, washing nets 20 times significantly reduced deterrence. Exiting and deterrence were also consistently higher with field-aged nets than washed nets, suggesting they elicited greater excitorepellency. Significantly lower proportions of mosquitoes were also collected inside field-aged nets (0–1%) compared to new unwashed (8–19%) and washed nets (23–33%) (*p* < 0.001), although this was probably due to their higher hole index (369–566) providing a greater surface area from which mosquitoes could escape.

**Table 1:** Entry, exiting and inside net results of wild, pyrethroid-resistant *Anopheles gambiae* sensu lato entering experimental huts in Covè, southern Benin

Net type	Net status	Mean hole index	*N* collected*	% Deterrence (95% CIs)	*N* inside veranda	% Exophily* (95% CIs)	*N* inside net	% Inside net* (95% CIs)
Untreated net (control)	–	138.0	882^a^	–	358	40.6^a^ (37.4–43.8)	346	39.2^a^ (36.0–32.4)
Interceptor^®^	New unwashed	138.0	753^b^	14.6 (5.6–26.3)	441	58.6^b^ (55.1–62.1)	144	19.1^bc^ (16.3–21.9)
	Washed 20 times	138.0	1522^c^	−72.6 (−83.5–61.7)	750	49.3^c^ (46.8–51.8)	498	32.7^d^ (30.3–35.1)
	Field-aged 3 years	454.2	706^de^	20.0 (11.1–28.9)	472	66.9^de^ (63.4–70.4)	1	0.1^e^ (0.0–0.3)
PermaNet^®^ 3.0	New unwashed	138.0	504^de^	42.9 (34.6–51.2)	378	75.0^f^ (71.2–78.8)	65	12.9^f^ (10.0–15.8)
	Washed 20 times	138.0	1292^cf^	−46.5 (−56.9–36.1)	845	65.4^d^ (62.8–68.0)	222	17.2^bc^ (15.1–19.3)
	Field-aged 3 years	369.3	823^b^	6.7 (−2.5–15.9)	589	71.6^ef^ (68.5–74.7)	9	1.1^ g^ (0.4–1.8)
Royal Guard^®^	New unwashed	138.0	1201^f^	−36.2 (−46.3–26.1)	883	73.5^f^ (71.0–76.0)	91	7.6^ h^ (6.1–9.1)
	Washed 20 times	138.0	1492^c^	−69.2 (−80.0–58.4)	879	58.9^b^ (56.4–61.4)	357	23.9^i^ (21.7–26.1)
	Field-aged 3 years	500.2	974^a^	−10.4 (−20.0–0.8)	729	74.8^f^ (72.1–77.5)	3	0.3^e^ (0.0–0.6)
Interceptor^®^ G2	New unwashed	138.0	828^be^	6.1 (−3.1–15.3)	521	62.9^bd^ (59.6–66.2)	135	16.3^b^ (13.8–18.8)
	Washed 20 times	138.0	1141^f^	−29.4 (−39.4–19.4)	670	58.7^b^ (55.8–66.2)	259	22.7^ci^ (20.3–25.1)
	Field-aged 3 years	566.2	850^be^	3.6 (−5.6–12.8)	587	69.1^de^ (55.8–61.6)	5	0.6^eg^ (0.1–1.1)

^*^Values in the same column bearing the same letter do not differ significantly at the 5% level, according to regression analysis. *CIs* = confidence intervals

#### Blood-feeding inhibition of wild pyrethroid-resistant malaria vectors

**Fig. 1:**
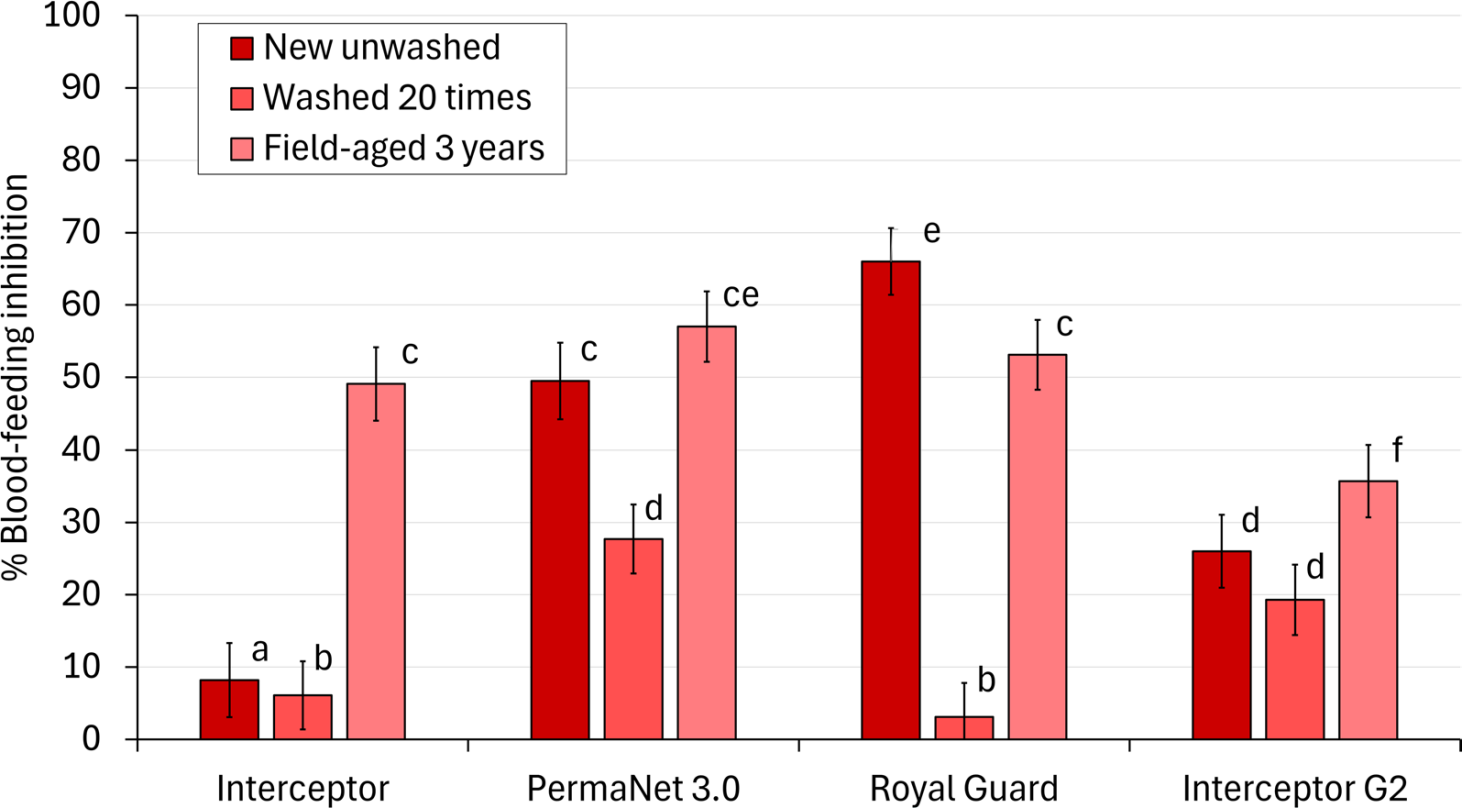
Blood-feeding inhibition of wild, pyrethroid-resistant *Anopheles gambiae* sensu lato entering experimental huts in Covè southern Benin. According to logistic regression, bars bearing the same letter do not differ at the 5% level. Errors bars represent 95% confidence intervals

The proportion of blood-fed *An. gambiae* s.l. with the untreated control net was 57%, and all ITN treatments significantly inhibited blood-feeding relative to the control except washed Interceptor® (6%, *p* = 0.189) and Royal Guard^®^ (3%, *p* = 0.247). While all unwashed next-generation ITNs outperformed unwashed Interceptor^®^, significantly higher blood-feeding inhibition was observed with Royal Guard^®^ (66%) compared to PermaNet^®^ 3.0 (50%, *p* < 0.001) and Interceptor^®^ G2 (26%, *p* < 0.001) (Fig. 1, Table 2). After 20 washes, however, blood-feeding inhibition with Royal Guard® fell to significantly lower levels than for PermaNet^®^ 3.0 (3% vs. 28%, *p* < 0.001) and Interceptor^®^ G2 (3% vs. 19%, *p* < 0.001) and similar to Interceptor^®^ (3% vs. 6%, *p* = 0.864). Between unwashed nets, PermaNet^®^ 3.0 provided superior blood-feeding protection than Interceptor^®^ G2 (50% vs. 26%, *p* < 0.001), but after 20 washes, there was no significant difference between these ITNs (28% vs. 19%, *p* = 0.207).

**Table 2:** Blood-feeding results of wild, pyrethroid-resistant *Anopheles gambiae* sensu lato entering experimental huts in Covè, southern Benin

Net type	Net status	Mean hole index	*N* collected*	*N* blood-fed	% Blood-feeding* (95% CIs)	% Blood-feeding inhibition (95% CIs)
Untreated net (control)	–	138.0	882^a^	506	57.4^a^ (54.1–60.7)	–
Interceptor®	New unwashed	138.0	753^b^	397	52.7^b^ (49.1–56.3)	8.2 (3.1–13.3)
	Washed 20 times	138.0	1522^c^	820	53.9^a^ (51.4–56.4)	6.1 (1.4–10.8)
	Field-aged 3 years	454.2	706^de^	206	29.2^c^ (25.8–32.6)	49.1 (44.1–54.1)
PermaNet® 3.0	New unwashed	138.0	504^de^	146	29.0^c^ (25.0–33.0)	49.5 (44.2–54.8)
	Washed 20 times	138.0	1292^cf^	536	41.5^d^ (38.8–44.2)	27.7 (22.9–32.5)
	Field-aged 3 years	369.3	823^b^	203	24.7^ce^ (21.8–27.6)	57.0 (52.1–61.9)
Royal Guard®	New unwashed	138.0	1201^f^	234	19.5^e^ (17.3–21.7)	66.0 (61.4–70.6)
	Washed 20 times	138.0	1492^c^	829	55.6^a^ (53.1–58.1)	3.1 (-1.6–7.8)
	Field-aged 3 years	500.2	974^a^	262	26.9^c^ (24.1–29.7)	53.1 (48.3–57.9)
Interceptor® G2	New unwashed	138.0	828^be^	352	42.5^d^ (39.1–45.9)	26.0 (21.0–31.0)
	Washed 20 times	138.0	1141^f^	528	46.3^d^ (43.4–49.2)	19.3 (14.4–24.2)
	Field-aged 3 years	566.2	850^be^	314	36.9^f^ (33.7–40.1)	35.7 (30.7–40.7)

^*^Values in the same column bearing the same letter do not differ significantly at the 5% level, according to regression analysis. *CIs* = confidence intervals

In contrast to the trend observed with new unwashed and washed nets, similar levels of blood-feeding inhibition were observed between field-aged Interceptor^®^ (49%), PermaNet^®^ 3.0 (57%) and Royal Guard^®^ (53%), and all of these nets significantly outperformed field-aged Interceptor^®^ G2 (36%). Blood-feeding inhibition was significantly lower with nets washed 20 times than with new, unwashed nets for all ITN types except Interceptor^®^ G2 (19% vs. 26%, *p* = 0.051). Unexpectedly, washing nets 20 times underestimated the end-of-life performance of all ITNs in terms of blood-feeding protection. Indeed, blood-feeding inhibition was significantly higher with field-aged nets compared to washed nets for Interceptor^®^ (49% vs. 6%, *p* < 0.001), PermaNet^®^ 3.0 (57% vs. 28%, *p* < 0.001), Royal Guard^®^ (53% vs. 3%, *p* < 0.001) and Interceptor^®^ G2 (36% vs. 19%, *p* < 0.001).

#### Mortality of wild pyrethroid-resistant malaria vectors

**Fig. 2:**
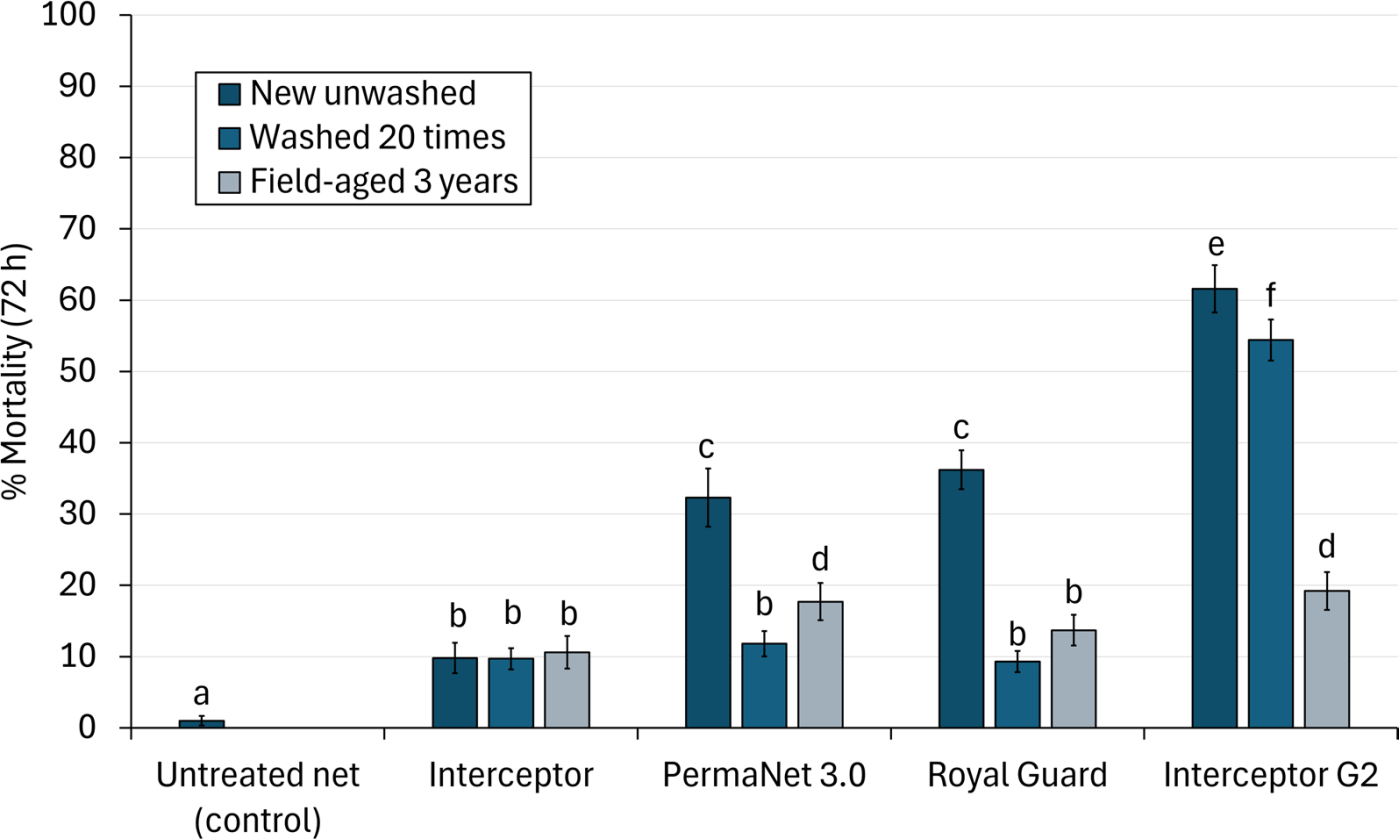
Mortality rates after 72 h of wild, pyrethroid-resistant *Anopheles gambiae* sensu lato entering experimental huts in Covè southern Benin. Bars bearing the same letter do not differ at the 5% level, according to logistic regression. Errors bars represent 95% confidence intervals

The proportion of dead mosquitoes after 72 h was 1% with the untreated control, and all ITN arms induced significantly higher mortality rates (*p* < 0.001). Interceptor^®^ G2 consistently killed higher proportions of mosquitoes than the other ITNs regardless of age, except with field-aged nets where mortality was similar to PermaNet^®^ 3.0 (19% vs. 18%, *p* = 0.797) (Fig. 2, Table 3). With new unwashed nets, PermaNet^®^ 3.0 and Royal Guard® outperformed Interceptor^®^ (32% and 36% vs. 10%, *p* < 0.001). However, this benefit was lost with washed and field-aged nets except for field-aged PermaNet^®^ 3.0, which provided a modest improvement in mortality compared to Interceptor^®^ (18% vs. 11%, *p* = 0.001).

**Table 3:** Mortality results of wild, pyrethroid-resistant *Anopheles gambiae* sensu lato entering experimental huts in Covè, southern Benin

Net type	Net status	Mean hole index	*N* collected*	*N* dead 72 h	% Mortality 72 h* (95% CIs)
Untreated net (control)	–	138.0	882^a^	9	1.0^a^ (0.3–1.7)
Interceptor^®^	New unwashed	138.0	753^b^	74	9.8^b^ (7.7–11.9)
	Washed 20 times	138.0	1522^c^	148	9.7^b^ (8.2–11.2)
	Field-aged 3 years	454.2	706^de^	75	10.6^b^ (8.3–12.9)
PermaNet^®^ 3.0	New unwashed	138.0	504^de^	163	32.3^c^ (28.2–36.4)
	Washed 20 times	138.0	1292^cf^	153	11.8^b^ (10.0–13.6)
	Field-aged 3 years	369.3	823^b^	146	17.7^d^ (15.1–20.3)
Royal Guard^®^	New unwashed	138.0	1201^f^	435	36.2^c^ (33.5–38.9)
	Washed 20 times	138.0	1492^c^	139	9.3^b^ (7.8–10.8)
	Field-aged 3 years	500.2	974^a^	133	13.7^b^ (11.5–15.9)
Interceptor^®^ G2	New unwashed	138.0	828^be^	510	61.6^e^ (58.3–64.9)
	Washed 20 times	138.0	1141^f^	621	54.4^f^ (51.5–57.3)
	Field-aged 3 years	566.2	850^be^	163	19.2^d^ (16.6–21.8)

^*^Values in the same column bearing the same letter do not differ significantly at the 5% level, according to regression analysis. *CIs* = confidence intervals

**Fig. 3:**
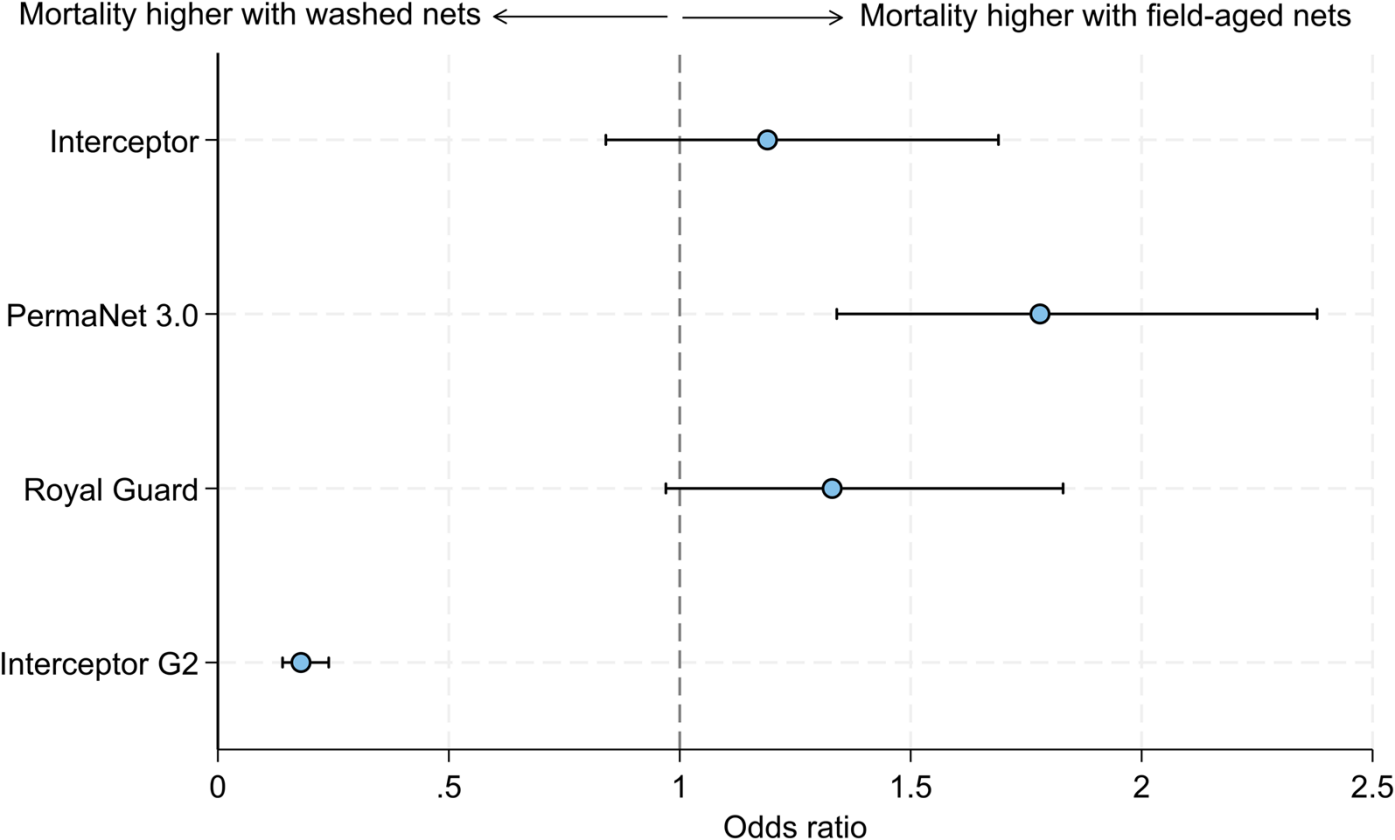
Odds ratios describing the difference in mortality rates after 72 h of wild, pyrethroid-resistant *Anopheles gambiae* sensu lato with nets washed 20 times to field-aged nets withdrawn from households 3 years post-distribution to nets washed 20 times. The dashed line represents an odds ratio of 1, indicating no difference in mortality between field-aged and washed nets. Odds ratios < 1 indicate higher mortality with washed nets. Error bars represent 95% confidence intervals

Washing nets significantly reduced mortality rates compared to new nets with all ITN types except Interceptor (10% vs. 10%, *p* = 0.575). Meanwhile, the ability of 20 washes to predict end-of-life killing performance varied depending on ITN type. Mortality was similar between washed and field-aged Interceptor^®^ (10% vs. 11%, *p* = 0.339, OR = 1.19, 95% CIs [0.84, 1.69]) and Royal Guard^®^ (9% vs. 14%, *p* = 0.076, OR = 1.33, 95% CIs [0.97, 1.83]), and although a statistically significant increase was detected with field-aged nets compared to washed nets with PermaNet^®^ 3.0 (18% vs. 12%, *p* < 0.001, OR = 1.78, 95% CIs [1.34, 2.38]), the absolute difference between arms was small. In contrast, mortality with field-aged Interceptor® G2 was significantly lower than for washed Interceptor^®^ G2 in absolute and statistical terms (19% vs. 54%, *p* < 0.001, OR = 0.18, 95% CIs [0.14, 0.24]) (Fig. 3).

#### Reduction in fertility of wild pyrethroid-resistant malaria vectors

**Fig. 4:**
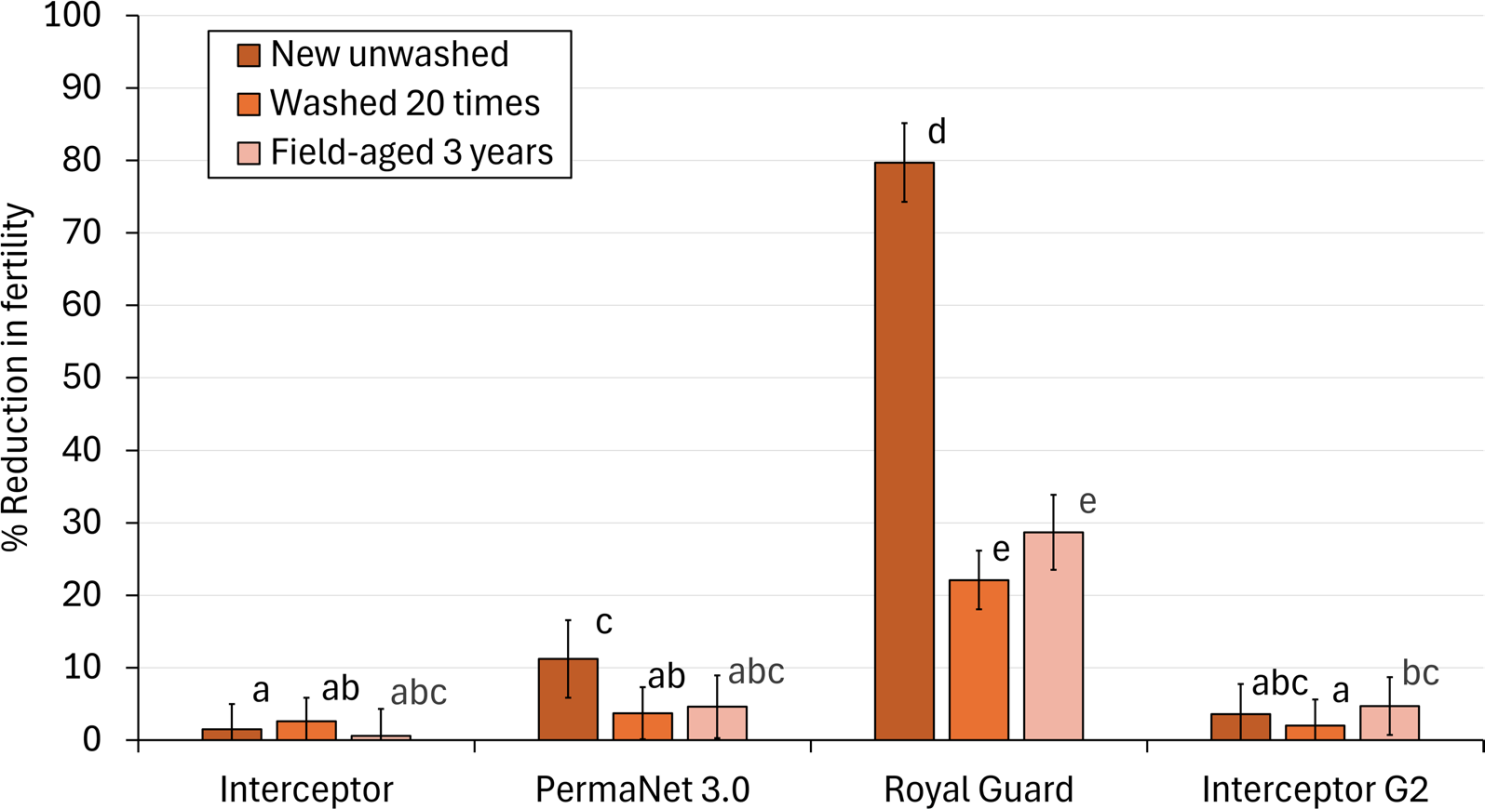
Reduction in fertility of wild, pyrethroid-resistant *Anopheles gambiae* sensu lato entering experimental huts in Covè southern Benin. Bars bearing the same letter do not differ at the 5% level according to logistic regression. Errors bars represent 95% confidence intervals

The proportion of fertile mosquitoes exposed to the untreated control net was 98%. Although some Interceptor^®^, PermaNet^®^ 3.0 and Interceptor^®^ G2 arms provided a statistically significant impact on fertility, this effect was negligible relative to the Royal Guard^®^ arms (Table 4). New unwashed Royal Guard^®^ induced the highest reduction in fertility of all treatment arms (80%); however, this fell significantly with washed nets (22%, *p* < 0.001) (Fig. 4). Reduction in fertility was similar between washed and field-aged Royal Guard^®^ (22% vs. 29%, *p* = 0.066), suggesting that washing 20 times provided a reasonable proxy for its end-of-life sterilising performance.

**Table 4:** Impact on fertility against wild, pyrethroid-resistant *Anopheles gambiae* sensu lato entering experimental huts in Covè, southern Benin

Net type	Net status	Mean hole index	*N* dissected	*N* fertile	% Fertility* (95% CIs)	% Reduction in fertility
Untreated net (control)	–	138.0	448	439	98.0^a^ (96.7–99.3)	–
Interceptor^®^	New unwashed	138.0	368	355	96.5^ab^ (94.6–98.4)	1.5 (−2.0–5.0)
	Washed 20 times	138.0	770	735	95.5^bc^ (94.0–97.0)	2.6 (−0.7–5.9)
	Field-aged 3 years	454.2	190	185	97.4^abcd^ (95.1–99.7)	0.6 (−3.1–4.3)
PermaNet^®^ 3.0	New unwashed	138.0	115	100	87.0^d^ (80.9–93.1)	11.2 (5.9–16.5)
	Washed 20 times	138.0	478	451	94.4^bc^ (92.3–96.5)	3.7 (0.1–7.3)
	Field-aged 3 years	369.3	186	174	93.5^abcd^ (90.0–97.0)	4.6 (0.3–8.9)
Royal Guard^®^	New unwashed	138.0	151	30	19.9^e^ (13.5–26.3)	79.7 (74.3–85.1)
	Washed 20 times	138.0	782	597	76.3^f^ (73.3–79.3)	22.1 (18.0–26.2)
	Field-aged 3 years	500.2	249	174	69.9^f^ (64.2–75.6)	28.7 (23.5–33.9)
Interceptor^®^ G2	New unwashed	138.0	201	190	94.5^bcd^ (91.3–97.7)	3.6 (−0.5–7.7)
	Washed 20 times	138.0	329	316	96.0^ab^ (93.9–98.1)	2.0 (−1.6–5.6)
	Field-aged 3 years	566.2	290	271	93.4^ cd^ (90.5–96.3)	4.7 (0.7–8.7)

^*^Values in the same column bearing the same letter do not differ significantly at the 5% level, according to regression analysis

### Supplementary laboratory bioassay results

**Fig. 5:**
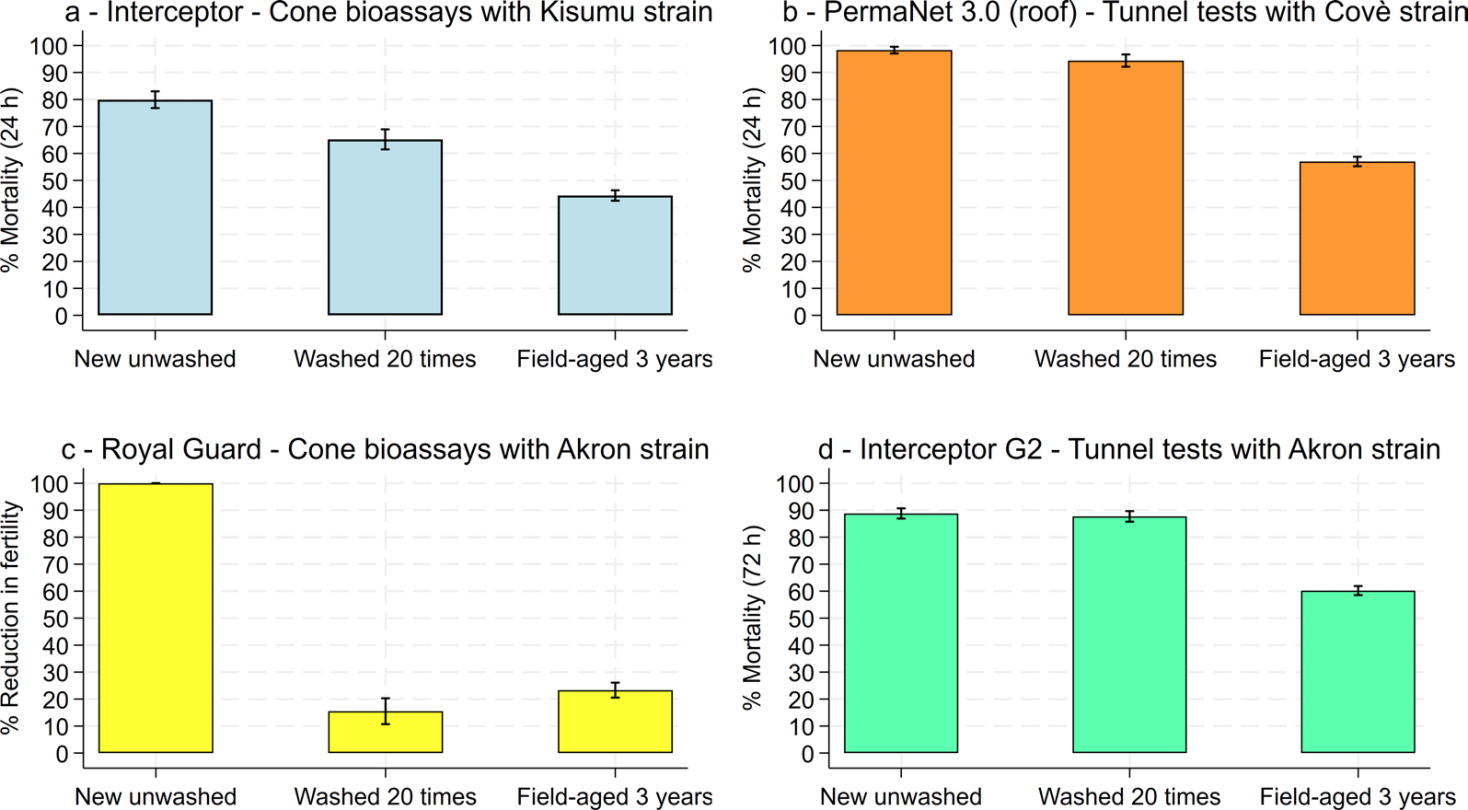
Supplementary laboratory bioassay results showing the bioavailability and potency of active ingredients on net pieces obtained from new unwashed nets, nets washed 20 times and nets withdrawn after 3 years of operational use. Each panel presents results with a different net type and test method. Error bars represent 95% confidence intervals

Net pieces cut from ITNs used in the experimental hut trial were tested in supplementary laboratory bioassays to characterise the bioavailability and potency of AIs on the surface of washed and field-aged nets. Cone bioassay results showed that washing nets reduced the potency of alpha-cypermethrin on Interceptor^®^ (90% mortality with unwashed nets vs. 65% mortality with nets washed 20 times) and PPF on Royal Guard^®^ (100% reduction in fertility with unwashed nets vs. 16% with nets washed 20 times) (Fig. 5). In contrast, mortality rates in tunnel tests were similar between unwashed and washed nets for PermaNet^®^ 3.0 (roof) (98% vs. 94%) and Interceptor^®^ G2 (89% vs. 88%).

The ability of 20 washes to simulate AI potency and bioavailability on field-aged nets varied between ITN types. Mortality was lower with field-aged nets than washed nets for Interceptor^®^ (65% vs. 44%), PermaNet^®^ 3.0 (roof) (94% vs. 57%) and Interceptor^®^ G2 (88% vs. 60%), suggesting that washing failed to simulate the loss of surface AI for these ITNs. In contrast, the sterilising effect of Royal Guard^®^ in cone bioassays was similar between washed and field-aged nets (16% vs. 23% reduction in fertility). This confirms the trend observed in experimental huts and indicates that the 20-wash method provided a suitable proxy for the potency of PPF on field-aged Royal Guard^®^. Mortality with the untreated control net was low in cone bioassays (1%) and tunnel tests (7%), while fertility in cone bioassays was high (97%), thus validating the tests. Full laboratory bioassay results are provided as supplementary information (Additional file 1: Tables S1 and S2).

### Chemical analysis results

**Fig. 6:**
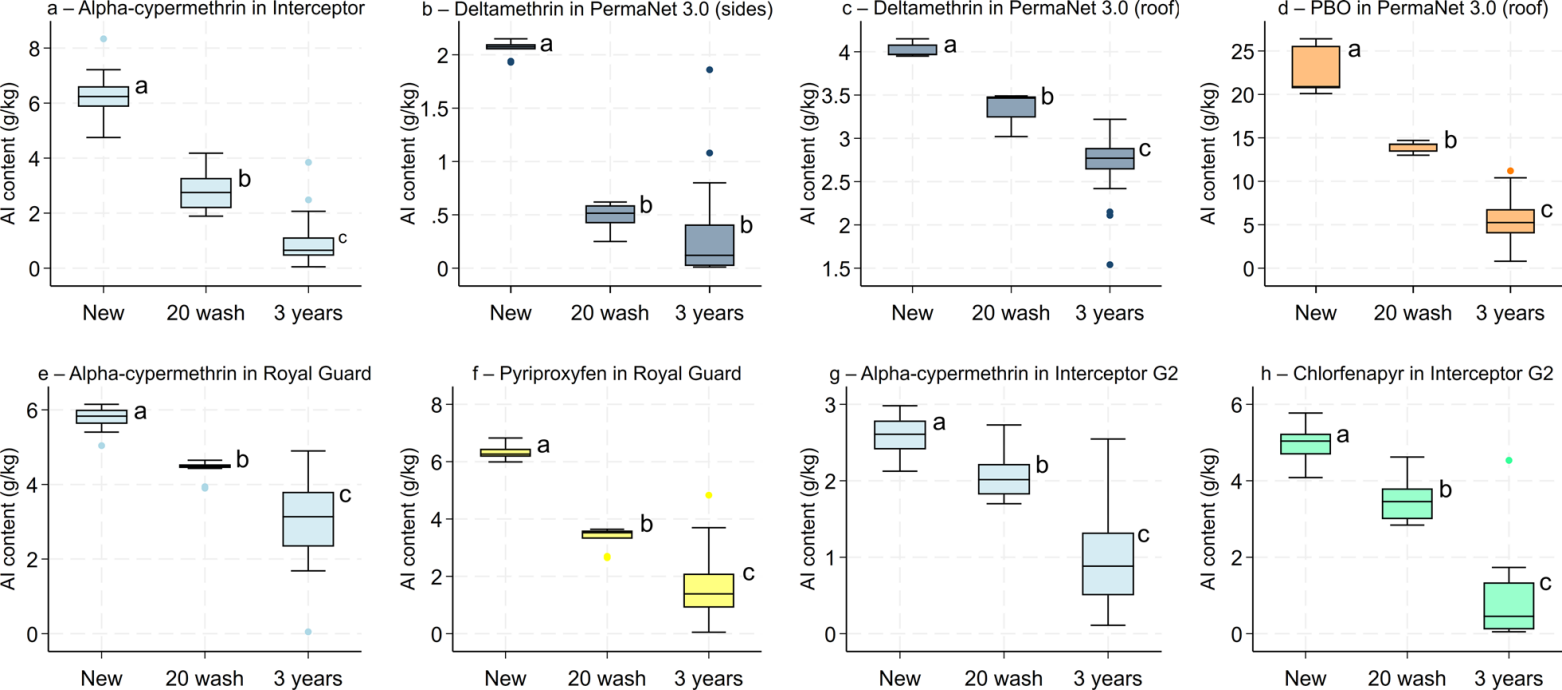
Chemical analysis results showing total active ingredient content (g/kg) in net pieces obtained from new unwashed nets, nets washed 20 times and nets withdrawn after 3 years of operational use. Each panel presents results with a different net type and active ingredient. In each panel, boxes bearing the same letter do not differ significantly at the 5% level, according to Tukey's honest significant difference post hoc tests

Net pieces cut from whole ITNs tested in experimental huts were sent for chemical analysis to assess whether washing nets 20 times simulated the loss of total AI content over 3 years of operational use. A one-way ANOVA revealed significant differences in total AI content (g/kg) between condition categories for all net types and AIs (*p* < 0.05). Post hoc Tukey’s tests revealed that washing 20 times significantly reduced AI content for all ITNs compared to new nets (*p* < 0.001) but broadly failed to simulate the loss of total AI content observed in field-aged nets. Indeed, total AI content was significantly lower (*p* < 0.001) with field-aged nets withdrawn after 3 years compared to nets washed 20 times across all net types and AIs except deltamethrin in PermaNet^®^ 3.0 (sides) (0.3 g/kg vs. 0.5 g/kg, *p* = 0.170) (Fig. 6).

**Fig. 7:**
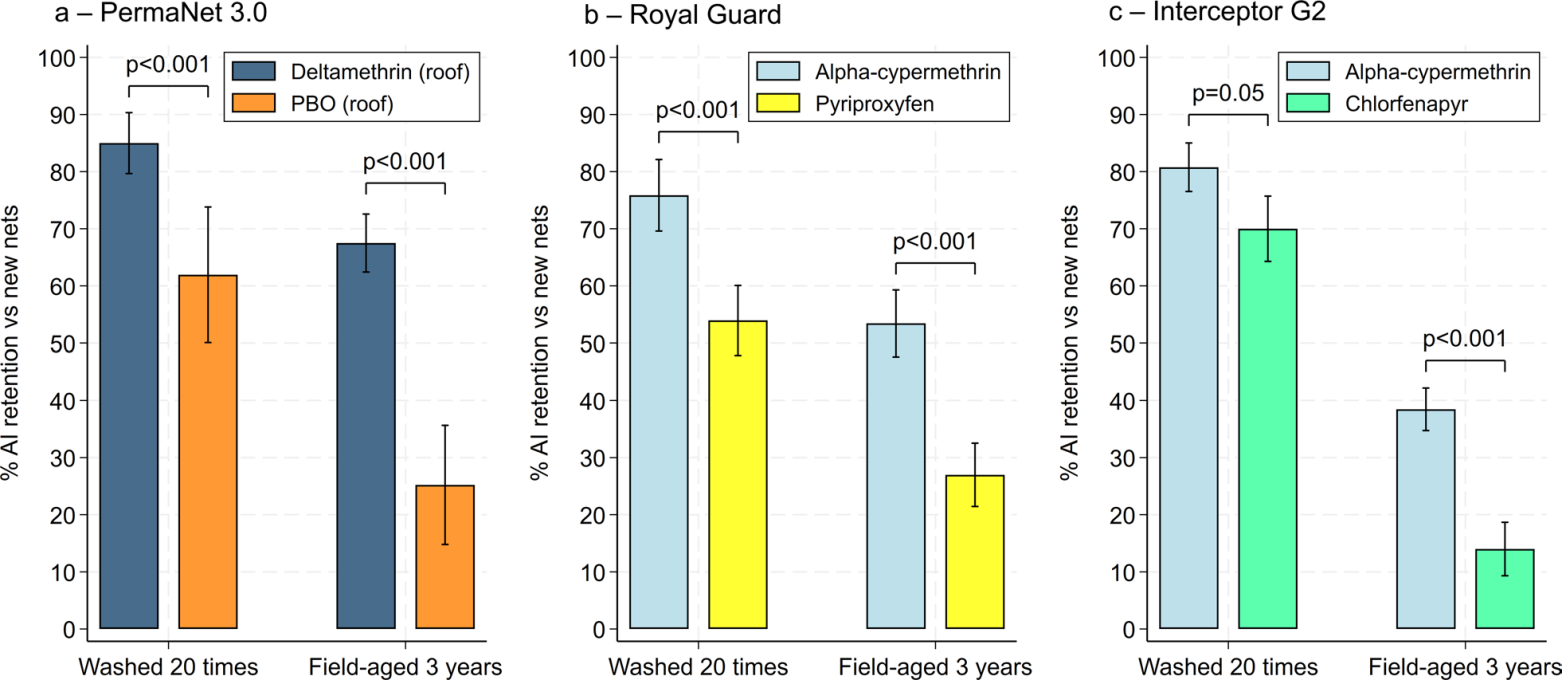
Proportional retention of the pyrethroid and non-pyrethroid components in washed and field-aged PermaNet® 3.0 (roof) (a), Royal Guard® (b) and Interceptor® G2 (c). Retention was calculated relative to the mean active ingredient content with new unwashed nets. Brackets between bars indicate pairwise comparisons between groups, with *p*-values representing the statistical significance. *P*-values < 0.05 were considered significant. Error bars represent 95% confidence intervals

For the next-generation nets, proportional retention of the pyrethroid and non-pyrethroid components were also compared separately with washed and field-aged nets. Retention of the non-pyrethroid was consistently lower than the pyrethroid regardless of net type and condition. With PermaNet^®^ 3.0 (roof) and Royal Guard^®^, this difference was statistically significant for both the washed nets (85% vs. 62%, *p* < 0.001 for PermaNet^®^ 3.0 (roof) and 76% vs. 54%, *p* < 0.001 for Royal Guard^®^) and the field-aged nets (68% vs. 25%, *p* < 0.001 for PermaNet^®^ 3.0 (roof) and 53% vs. 27%, *p* < 0.001 for Royal Guard^®^). With Interceptor^®^ G2, however, retention of CFP compared to alpha-cypermethrin was significantly lower with the field-aged nets (39% vs. 14%, *p* < 0.001) but similar with the washed nets (81% vs. 70%, *p* = 0.05) (Fig. 7). Full chemical analysis results are provided as supplementary information (Additional file 1: Table S3).

### Susceptibility bioassay results

**Fig. 8:**
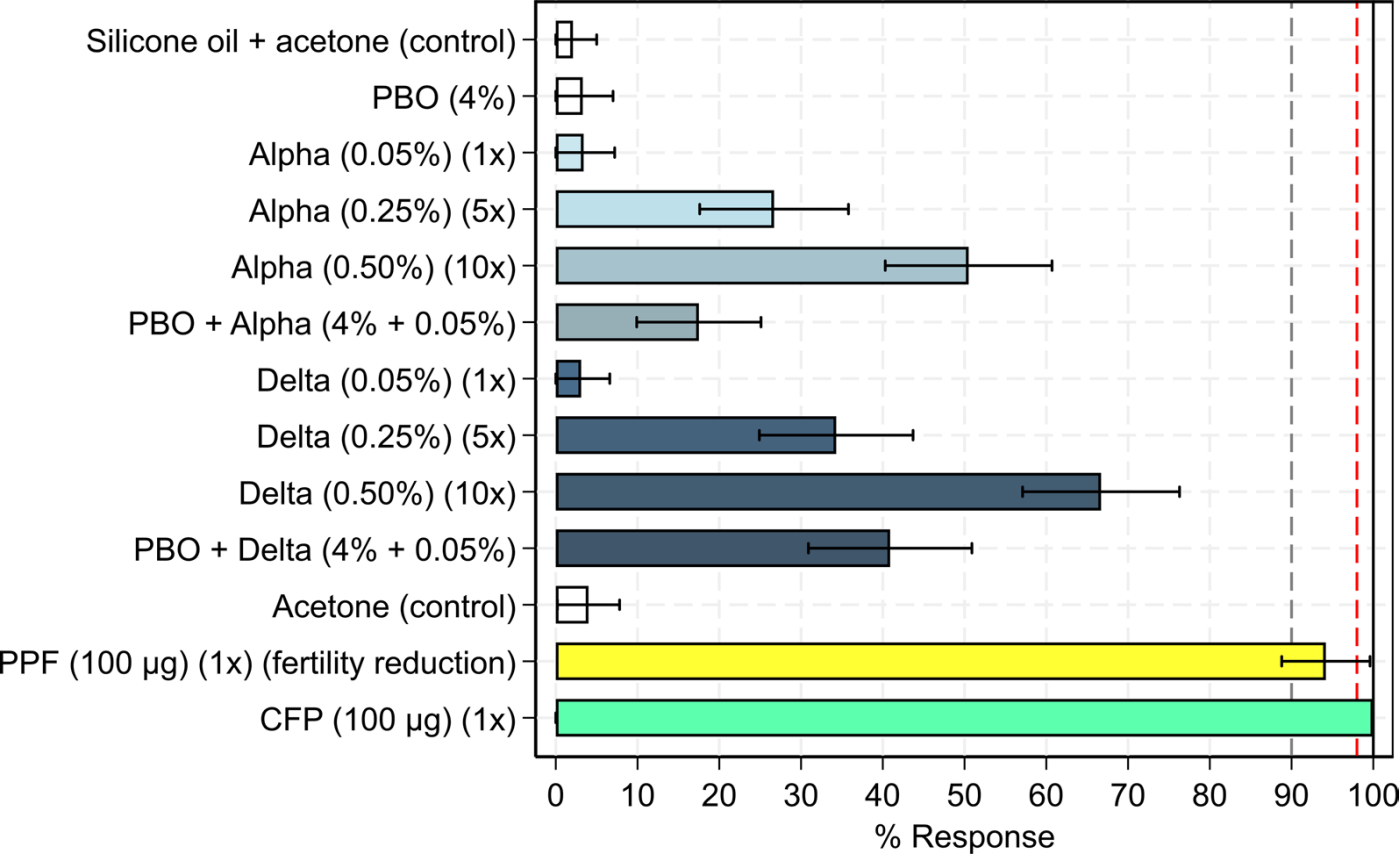
Susceptibility bioassay results with wild *Anopheles gambiae* sensu lato collected near the experimental hut site in Covè, southern Benin. Mortality was recorded after 24 h for pyrethroid exposures and after 72 h for chlorfenapyr. Pyriproxyfen susceptibility was assessed based on the reduction in fertility of surviving blood-fed mosquitoes relative to the untreated control observed via ovary dissection. Red dashed line represents the standard 98% susceptibility cut-off, while the grey dashed line represents the provisional 90% cut-off to confirm chlorfenapyr resistance. Error bars represent 95% confidence intervals

Mortality with the discriminating dose of alpha-cypermethrin and deltamethrin was negligible (3% with both), demonstrating the near ubiquitous levels of pyrethroid resistance in the Covè vector population (Fig. 8). Mortality increased progressively with 5 × and 10 × the discriminating doses of alpha-cypermethrin (27% with 5 × and 51% with 10 ×) and deltamethrin (34% with 5 × and 67% with 10 ×), indicating high-intensity pyrethroid resistance. Pre-exposure to the P450 inhibitor PBO improved the mortality response to the discriminating dose of both insecticides (18% vs. 3% and 41% vs. 3%, respectively) but failed to restore full susceptibility, suggesting that P450s were partially responsible for pyrethroid resistance. The discriminating concentration of CFP induced 100% mortality, demonstrating continued susceptibility. The reduction in fertility with the discriminating concentration of PPF was also high (94%), suggesting the population remained largely susceptible to the growth regulator. Full susceptibility bioassay results are provided as supplementary information (Additional file 1: Tables S4 and S5).

## Discussion

Nets are washed 20 times as part of experimental hut evaluations to simulate the loss of AI in ITNs over 3 years of operational use and assess their insecticidal durability. Although the 20-wash method is a standard part of experimental hut trials of ITNs, which are integral to WHO regulatory processes, its ability to predict the end-of-life performance of ITNs has not been robustly empirically validated. In this study, we compared the entomological performance and chemical content of ITNs washed 20 times to field-aged ITNs withdrawn from households 3 years post-distribution against a pyrethroid-resistant vector population in an experimental hut trial in Benin. The results showed that while the WHO wash method was predictive of the end-of-life sterilising performance of Royal Guard®, its ability to forecast mosquito mortality depended on ITN type. Unexpectedly, we also found that washing consistently underestimated the levels of blood-feeding protection provided by field-aged nets.

The 20-wash method provided a reasonable proxy for the end-of-life killing and sterilising performance of Interceptor®, PermaNet® 3.0 and Royal Guard® in experimental huts. Washed nets induced similar mortality rates to field-aged nets for Interceptor® and Royal Guard®, and although a statistically significant increase in mortality was detected with field-aged nets over washed nets for PermaNet^®^ 3.0, the absolute difference between study arms was small. Reduction in fertility with Royal Guard^®^—whose PPF component sterilises mosquitoes—was also similar between washed and field-aged nets, supporting the value of washing nets 20 times. Despite their similar performance in experimental huts, total AI content and surface bioavailability were generally lower with field-aged nets than washed nets. The high levels of pyrethroid resistance at the Covè hut site could also have masked potential differences in mortality, which may have been more evident against a more susceptible vector population.

In contrast to the other ITNs, mortality was significantly higher with washed nets than field-aged nets for Interceptor^®^ G2 (54% vs. 19%), suggesting that washing provided a poor proxy for its end-of-life performance. This difference in mortality between washed and field-aged Interceptor^®^ G2 is attributable to the lower retention and surface availability of CFP with field-aged nets (15% vs. 58%), as demonstrated by the chemical analysis and supplementary bioassays. This corroborates findings from recent durability studies showing a substantial loss in the bioefficacy of Interceptor^®^ G2 nets after 3 years of household use when tested in laboratory bioassays [38] and experimental huts [39]. Despite this, previous experimental hut trials in Benin [32, 33], Burkina Faso [34], Côte d’Ivoire [35], Kenya [36] and Tanzania [37] have reported similar mortality with Interceptor^®^ G2 and other pyrethroid-CFP nets between unwashed nets and nets washed 20 times, indicating good wash resistance and insecticidal durability. However, the results of this study suggest that by failing to simulate the total loss of CFP bioavailability over 3 years of operational use, 20 washes may overestimate the end-of-life performance of Interceptor^®^ G2 in Benin, thereby biasing efficacy estimates from hut trials. This may also explain why Interceptor^®^ G2 lost its improved impact on malaria compared to a pyrethroid-only net in the 3rd year of the recent cluster RCT in Benin [14] despite previous hut trials indicating good wash resistance and insecticidal durability. These findings suggest that a higher number of washes or alternative artificial ageing techniques may be needed to generate more predictive estimates of entomological efficacy for pyrethroid-CFP nets. Field-aged nets could also be used for hut trials; however, the time and cost implications must be carefully considered.

Levels of blood-feeding inhibition were consistently higher with field-aged nets compared to nets washed 20 times across all ITN types. The personal protective benefit of ITNs is attributable to the physical barrier provided by the net and the excitorepellency of the pyrethroid. Since hole index was higher and pyrethroid content was lower with field-aged nets compared to washed nets, their superior impact on mosquito blood-feeding was unexpected. Recent experimental hut trials were performed at the same hut site in Benin to assess the entomological performance of the same ITN brands withdrawn from households after 12, 24 and 36 months to detect changes in bioefficacy over time. The results mirror this study, showing that blood-feeding inhibition with field-aged nets rose steadily with increasing time elapsed from distribution up to 24 months and that nets withdrawn at 36 months provided similar or superior blood-feeding protection compared to new nets [39]. Field-aged nets also induced higher deterrence and exophily in the present trial, suggesting they elicited greater excitorepellency than their washed counterparts. These findings indicate that the superior personal protection with the field-aged nets may be due to contamination with repellent compounds inside households. This could arise from the concurrent use of domestic pesticidal products such as mosquito coils or sprays or potentially local retreatment of nets, although there is currently no evidence to confirm this. In contrast to this study, previous hut trials comparing the efficacy of new nets to those withdrawn from households in Burkina Faso [40] and Tanzania [41] did not demonstrate enhanced blood-feeding protection with the field-aged nets. This highlights the need for further research to investigate the reasons for this phenomenon and its generalisability across different settings.

Care must be taken not to overgeneralise the findings of this study, as the efficacy of field-aged ITNs in experimental huts will likely depend on the community from which they are obtained. Self-reported data from household surveys conducted in the study area as part of the larger ITN durability monitoring study [29] indicated that users washed their nets approximately six times on average over 3 years, corresponding to a wash frequency of twice per year. Prior studies show, however, that ITN care and use practices vary widely between different communities [42]. In Iran [43], Uganda [44] and Zambia [45], users reported washing nets on average a similar number of times over 3 years to the present study. However, other studies in Mali [46], Senegal [47], Tanzania [48] and Uganda [49] found considerably higher wash frequencies, with some users washing their nets up to eight times a month due to social norms around cleanliness. This is complicated further by variations in washing methods, which may be correlated with greater reductions in AI and entomological efficacy, including drying nets in the sun and using harsh detergents or more abrasive washing techniques [20, 47]. Further studies investigating differences in ITN washing practices and how this affects the entomological performance of field-aged ITNs across multiple settings spanning a range of cultural and environmental contexts are advisable.

## Conclusions

In this setting, the 20-wash method was predictive of the end-of-life performance of the study ITNs regarding their sterilising effects, whereas, for the endpoints of mosquito mortality and blood-feeding inhibition, the predictive value was either inconsistent or limited across different ITN types. Mortality rates were generally similar or slightly higher with field-aged pyrethroid-only (Interceptor^®^), pyrethroid-PBO (PermaNet^®^ 3.0) and pyrethroid-PPF (Royal Guard^®^) nets compared to washed nets, suggesting that washing 20 times provided a reasonable proxy for the end-of-life killing performance of these nets. In contrast, washed pyrethroid-CFP nets (Interceptor^®^ G2) induced significantly higher mortality than their field-aged counterparts because of a greater loss of CFP in the field-aged nets. The WHO wash method may thus overestimate the end-of-life performance of Interceptor^®^ G2 in this setting, potentially biasing efficacy estimates from experimental hut trials. Field-aged nets unexpectedly outperformed washed nets in terms of blood-feeding inhibition despite their lower pyrethroid content and higher hole index. Further research is needed to understand the superior blood-feeding protection observed with field-aged nets and assess the predictive value of the WHO wash method for field-aged ITNs withdrawn from other communities with different net care and use practices.

## Supplementary Information

Additional file 1.

## Data Availability

All data supporting the findings of this study are available within the paper and its supplementary material.

## Abbreviations

AI: Active ingredient
CFP: Chlorfenapyr
CI: Confidence interval
ITN: Insecticide-treated nets
*Kdr*: Knockdown resistance
LSHTM: London School of Hygiene & Tropical Medicine
PBO: Piperonyl butoxide
PPF: Pyriproxyfen
PQT/VCP: Prequalification unit vector control product assessment team
P450: Cytochrome P450 monooxygenase
OR: Odds ratio
RCT: Randomised controlled trial
WHO: World Health Organisation
