# Supplementary material for: Does washing insecticide-treated nets 20 times for experimental hut evaluations provide a suitable proxy for their end-of-life performance under household conditions?
Source: Parasit Vectors. 2025 Apr 21;18:148. doi: 10.1186/s13071-025-06743-w (PMC12010526; doi:10.1186/s13071-025-06743-w)
Supplement: Supplementary file 1 — Additional file 1. [file 13071_2025_6743_MOESM1_ESM.docx]

**Supplementary information**

**Table S1** Supplementary cone bioassay results with unfed susceptible *Anopheles gambiae* sensu stricto Kisumu strain to characterise the bioavailability of alpha-cypermethrin on Interceptor® and blood-fed insecticide-resistant *Anopheles coluzzii* Akron strain to characterise the bioavailability of pyriproxyfen on Royal Guard®. *CIs=confidence intervals.*

| **Strain** | ***Anopheles gambiae* sensu stricto Kisumu** | | | | ***Anopheles coluzzii* Akron** | | | |
| --- | --- | --- | --- | --- | --- | --- | --- | --- |
| **Feeding status** | **Unfed** | | | | **Blood-fed** | | | |
| **Net type** | **Untreated net (control)** | **Interceptor** | | | **Untreated net (control)** | **Royal Guard** | | |
| **Status** | **̶** | **New unwashed** | **New washed 20 times** | **Field-aged 3 years** | **̶** | **New unwashed** | **New washed 20 times** | **Field-aged 3 years** |
| ***N* exposed** | 520 | 638 | 632 | 2538 | 1174 | 704 | 728 | 2480 |
| ***N* KD 60 mins** | 2 | 584 | 578 | 1963 | 0 | 612 | 388 | 872 |
| **% KD 60 mins** | 0.4 | 91.5 | 91.5 | 77.3 | 0 | 86.9 | 53.3 | 35.2 |
| **95% CIs** | 0.0-0.9 | 89.3-93.7 | 89.3-93.7 | 75.7-78.9 | ̶ | 84.4-89.4 | 49.7-56.9 | 33.3-37.1 |
| ***N* dead 24 h** | 6 | 510 | 412 | 1126 | 2 | 510 | 142 | 320 |
| **% Mortality 24 h** | 1.2 | 79.9 | 65.2 | 44.4 | 0.2 | 72.4 | 19.5 | 12.9 |
| **95% CIs** | 0.3-2.1 | 76.8-83.0 | 61.5-68.9 | 42.5-46.3 | 0.0-0.5 | 69.1-75.7 | 16.6-22.4 | 11.6-14.2 |
| ***N* dead 48 h** | ̶ | ̶ | ̶ | ̶ | 12 | 558 | 186 | 441 |
| **% Mortality 48 h** | ̶ | ̶ | ̶ | ̶ | 1.0 | 79.3 | 25.5 | 17.8 |
| **95% CIs** | ̶ | ̶ | ̶ | ̶ | 0.4-1.6 | 76.3-82.3 | 22.3-28.7 | 16.3-19.3 |
| ***N* dead 72 h** | ̶ | ̶ | ̶ | ̶ | 28 | 580 | 222 | 539 |
| **% Mortality 72 h** | ̶ | ̶ | ̶ | ̶ | 2.4 | 82.4 | 30.5 | 21.7 |
| **95% CIs** | ̶ | ̶ | ̶ | ̶ | 1.5-3.3 | 79.6-85.2 | 27.2-33.8 | 20.1-23.3 |
| ***N* dissected** | ̶ | ̶ | ̶ | ̶ | 394 | 64 | 245 | 950 |
| ***N* fertile** | ̶ | ̶ | ̶ | ̶ | 382 | 0 | 201 | 707 |
| **% Fertility** | ̶ | ̶ | ̶ | ̶ | 97.0 | 0 | 82.0 | 74.4 |
| **95% CIs** | ̶ | ̶ | ̶ | ̶ | 95.3-98.7 | ̶ | 77.2-86.8 | 71.6-77.2 |
| **% Reduction in fertility** | ̶ | ̶ | ̶ | ̶ | ̶ | 100 | 15.5 | 23.3 |
| **95% CIs** | ̶ | ̶ | ̶ | ̶ | ̶ | ̶ | 10.5-20.5 | 19.2-27.4 |

**Table S2** Supplementary tunnel test results with the insecticide-resistant *Anopheles gambiae* sensu lato Covè and *Anopheles coluzzii* Akron strains to characterise the bioavailability of piperonyl butoxide on PermaNet® 3.0 (roof) and chlorfenapyr on Interceptor® G2. *CIs=confidence intervals.*

| **Strain** | ***Anopheles gambiae* sensu lato Covè** | | | | ***Anopheles coluzzii* Akron** | | | |
| --- | --- | --- | --- | --- | --- | --- | --- | --- |
| **Net type** | **Untreated net (control)** | **PermaNet 3.0 (roof)** | | | **Untreated net (control)** | **Interceptor G2** | | |
| **Status** | **̶** | **New unwashed** | **New washed 20 times** | **Field-aged 3 years** | **̶** | **New unwashed** | **New washed 20 times** | **Field-aged 3 years** |
| ***N* exposed** | 777 | 413 | 393 | 3054 | 1129 | 1067 | 1077 | 3218 |
| ***N* passage** | 654 | 125 | 177 | 1290 | 950 | 414 | 568 | 2034 |
| **% Passage** | 84.2 | 30.3 | 45.0 | 42.2 | 84.1 | 38.8 | 52.7 | 63.2 |
| **95% CIs** | 81.6-86.8 | 25.9-34.7 | 40.1-49.9 | 40.4-44.0 | 82.0-86.2 | 35.9-41.7 | 49.7-55.7 | 61.5-64.9 |
| ***N* blood-fed** | 615 | 4 | 16 | 439 | 869 | 265 | 348 | 1512 |
| **% Blood-feeding** | 79.2 | 1.0 | 4.1 | 14.4 | 77.0 | 24.8 | 32.3 | 47.0 |
| **95% CIs** | 76.3-82.1 | 0.0-2.0 | 2.1-6.1 | 13.2-15.6 | 74.5-79.5 | 22.2-27.4 | 29.5-35.1 | 45.3-48.7 |
| **% Blood-feeding inhibition** | ̶ | 98.7 | 94.8 | 81.8 | ̶ | 67.8 | 58.1 | 39.0 |
| **95% CIs** | ̶ | 94.9-100 | 90.5-99.1 | 77.8-85.8 | ̶ | 63.4-72.2 | 53.6-62.5 | 35.0-43.0 |
| ***N* dead immediate** | 44 | 397 | 346 | 1589 | 20 | 638 | 553 | 936 |
| **% Immediate mortality** | 5.7 | 96.1 | 88.0 | 52.0 | 1.8 | 59.8 | 51.3 | 29.1 |
| **95% CIs** | 4.1-7.3 | 94.2-98.0 | 84.8-91.2 | 50.2-53.8 | 1.0-2.6 | 56.9-62.7 | 48.3-54.3 | 27.5-30.7 |
| ***N* dead 24 h** | 45 | 406 | 371 | 1740 | 30 | 903 | 875 | 1688 |
| **% Mortality 24 h** | 5.8 | 98.3 | 94.4 | 57.0 | 2.7 | 84.6 | 81.2 | 52.5 |
| **95% CIs** | 4.2-7.4 | 97.1-99.5 | 92.1-96.7 | 55.2-58.8 | 1.8-3.6 | 82.4-86.8 | 78.9-83.5 | 50.8-54.2 |
| ***N* dead 48 h** | ̶ | ̶ | ̶ | ̶ | 43 | 924 | 911 | 1845 |
| **% Mortality 48 h** | ̶ | ̶ | ̶ | ̶ | 3.8 | 86.6 | 84.6 | 57.3 |
| **95% CIs** | ̶ | ̶ | ̶ | ̶ | 2.7-4.9 | 84.6-88.6 | 82.4-86.8 | 55.6-59.0 |
| ***N* dead 72 h** | ̶ | ̶ | ̶ | ̶ | 79 | 948 | 944 | 1938 |
| **% Mortality 72 h** | ̶ | ̶ | ̶ | ̶ | 7.0 | 88.8 | 87.7 | 60.2 |
| **95% CIs** | ̶ | ̶ | ̶ | ̶ | 5.5-8.5 | 86.9-90.7 | 85.7-89.7 | 58.5-61.9 |

**Table S3** Chemical analysis results showing total active ingredient content (g/kg) in net pieces cut from whole Interceptor®, PermaNet® 3.0, Royal Guard® and Interceptor® nets when new unwashed, washed 20 times and after 3 years of operational use. **Values for the same net type and active ingredient bearing the same letter do not differ significantly at the 5% level according to Tukey's Honest Significant Difference post-hoc tests. CIs=confidence intervals.*

| **Net type** | **Active ingredient (AI)** | **Condition** | **Mean AI content g/kg (95% CIs)*** | **% Relative standard deviation** | **% AI retention vs. new nets**  **(95% CIs)** |
| --- | --- | --- | --- | --- | --- |
| Interceptor | Alpha-cypermethrin | New unwashed | 6.3^a^ (6.0-6.5) | 10.9 | ̶ |
|  |  | Washed 20 times | 2.8^b^ (2.2-3.3) | 26.6 | 44.4 (38.5-50.4) |
|  |  | Field-aged 3 years | 0.9^c^ (0.6-1.2) | 89.9 | 14.3 (9.0-19.5) |
| PermaNet 3.0 | Deltamethrin (sides) | New unwashed | 2.1^a^ (2.0-2.1) | 3.5 | ̶ |
|  |  | Washed 20 times | 0.5^b^ (0.4-0.6) | 28.4 | 23.8 (20.6-27.0) |
|  |  | Field-aged 3 years | 0.3^b^ (0.1-0.4) | 149.7 | 14.3 (11.2-17.3) |
|  | Deltamethrin (roof) | New unwashed | 4.0^a^ (4.0-4.1) | 1.9 | ̶ |
|  |  | Washed 20 times | 3.4^b^ (3.2-3.5) | 6.2 | 85.0 (79.7-90.3) |
|  |  | Field-aged 3 years | 2.7^c^ (2.6-2.8) | 12.7 | 67.5 (64.2-72.6) |
|  | Piperonyl butoxide (roof) | New unwashed | 22.6^a^ (20.3-24.9) | 12.0 | ̶ |
|  |  | Washed 20 times | 14.0^b^ (13.4-14.5) | 4.4 | 61.9 (50.1-73.8) |
|  |  | Field-aged 3 years | 5.7^c^ (4.7-6.6) | 45.9 | 25.2 (14.8-35.6) |
| Royal Guard | Alpha-cypermethrin | New unwashed | 5.8^a^ (5.7-5.9) | 4.4 | ̶ |
|  |  | Washed 20 times | 4.4^b^ (4.3-4.6) | 5.4 | 75.9 (69.6-82.1) |
|  |  | Field-aged 3 years | 3.1^c^ (2.7-3.5) | 33.5 | 53.4 (47.6-59.3) |
|  | Pyriproxyfen | New unwashed | 6.3^a^ (6.2-6.4) | 3.0 | ̶ |
|  |  | Washed 20 times | 3.4^b^ (3.2-3.6) | 10.2 | 54.0 (47.9-60.1) |
|  |  | Field-aged 3 years | 1.7^c^ (1.3-2.1) | 66.1 | 27.0 (21.4-32.5) |
| Interceptor G2 | Alpha-cypermethrin | New unwashed | 2.6^a^ (2.5-2.7) | 8.7 | ̶ |
|  |  | Washed 20 times | 2.1^b^ (1.9-2.2) | 14.5 | 80.8 (76.5-85.0) |
|  |  | Field-aged 3 years | 1.0^c^ (0.8-1.2) | 60.0 | 38.5 (34.7-42.2) |
|  | Chlorfenapyr | New unwashed | 5.0^a^ (4.8-5.1) | 8.7 | ̶ |
|  |  | Washed 20 times | 3.5^b^ (3.1-3.8) | 15.2 | 70.0 (64.3-75.7) |
|  |  | Field-aged 3 years | 0.7^c^ (0.4-1.1) | 123.9 | 14.0 (9.3-18.7) |

**Tables S4** Mortality results of wild pyrethroid-resistant *Anopheles gambiae* sensu lato in World Health Organisation tube tests and bottle bioassays. *Mortality was recorded after 24 h for pyrethroid exposures and after 72 h for chlorfenapyr and pyriproxyfen. CIs=confidence intervals.*

| **Insecticide** | **Dose** | ***N* exposed** | ***N* dead** | **% Mortality**  **(95% CIs)** |
| --- | --- | --- | --- | --- |
| Silicone oil + acetone (control) | ̶ | 97 | 2 | 2.1  (0.0-5.0) |
| Piperonyl butoxide | 4% | 92 | 3 | 3.3  (0.0-7.0) |
| Alpha-cypermethrin | 0.05% (1x) | 89 | 3 | 3.4  (0.0-7.2) |
|  | 0.25% (5x) | 90 | 24 | 26.7  (17.6-35.8) |
|  | 0.50% (10x) | 93 | 47 | 50.5  (40.3-60.7) |
| Piperonyl butoxide + Alpha-cypermethrin | 4% + 0.05% | 97 | 17 | 17.5  (9.9-25.1) |
| Deltamethrin | 0.05% (1x) | 96 | 3 | 3.1  (0.0-6.6) |
|  | 0.25% (5x) | 99 | 34 | 34.3  (24.9-43.7) |
|  | 0.50% (10x) | 93 | 62 | 66.7  (57.1-76.3) |
| Piperonyl butoxide + Deltamethrin | 4% + 0.05% | 93 | 38 | 40.9  (30.9-50.9) |
| Acetone (control) | ̶ | 100 | 4 | 4.0  (0.2-7.8) |
| Chlorfenapyr | 100 µg | 100 | 100 | 100  (–) |
| Acetone (control) | ̶ | 95 | 0 | 0.0  (–) |
| Pyriproxyfen | 100 µg | 88 | 5 | 5.7  (0.3-11.1) |

**Table S5** Fertility results of wild blood-fed pyrethroid-resistant *Anopheles gambiae* sensu lato in World Health Organisation bottle bioassays to assess pyriproxyfen susceptibility. *A subsample of mosquitoes surviving after the 72 h mortality recording period was dissected to score ovary development and score fertility.* *CIs=confidence intervals.*

| **Insecticide** | **Dose** | ***N* exposed** | ***N* dissected** | ***N* fertile** | **% Fertility**  **(95% CIs)** | **% Reduction in fertility**  **(95% CIs)** |
| --- | --- | --- | --- | --- | --- | --- |
| Acetone (control) | ̶ | 95 | 87 | 86 | 98.9  (96.7-100) | ̶ |
| Pyriproxyfen | 100 µg | 88 | 70 | 4 | 5.7  (0.3-11.1) | 94.2  (88.8-99.6) |
